# Association Between Natural/Built Campus Environment and Depression Among Chinese Undergraduates: Multiscale Evidence for the Moderating Role of Socioeconomic Factors After Controlling for Residential Self-Selection

**DOI:** 10.3389/fpubh.2022.844541

**Published:** 2022-04-07

**Authors:** Haoran Yang, Xiangfen Cui, Martin Dijst, Senlin Tian, Jie Chen, Jianhong Huang

**Affiliations:** ^1^The Centre for Modern Chinese City Studies, Research Center for China Administrative Division, Future City Lab, East China Normal University, Shanghai, China; ^2^Faculty of Environmental Science and Engineering, Kunming University of Science and Technology, Kunming, China; ^3^Department of Urban Development and Mobility, Luxembourg Institute of Socio-Economic Research (LISER), Esch-sur-Alzette, Luxembourg; ^4^Faculty of Land Resource Engineering, Kunming University of Science and Technology, Kunming, China

**Keywords:** natural environment, built environment, multiple scales, depressive symptoms, moderating effect, undergraduates, China

## Abstract

**Aim:**

Evidence on the association between natural-built environments and depression is largely derived from the general population and prone to residential self-selection bias because of the nature of cross-sectional research design. Despite emerging adulthood, which includes the university years, is a critical stage for forming life-long health habits, studies on this topic focusing on undergraduate students are limited. The current study aims to illustrate the underlying mechanisms for how the campus-based environments affect depression in undergraduate students.

**Methods:**

Based on a nationwide representative analytical sample of 22,009 Chinese undergraduates in 2018, we examined participants' reports of depression and campus-centered natural/built environments within multiple buffer sizes including 0.5, 1.0, and 2.5 km. After disentangling residential self-selection, we explored the moderating role of the socioeconomic attributes of undergraduates. The depression outcome was measured by the nine-item Patient Health Questionnaire (PHQ9). Indicators of exposure to green and blue space, transportation infrastructure, and food environments were objectively assessed using different circular buffers around each campus address.

**Results:**

Modeling results indicated that campus neighborhoods with more scattered trees (0.5 km), water (0.5, 1.0, and 2.5 km), and street intersections (1.0 and 2.5 km) were protective against depression. In contrast, those living near denser distributions of outlets serving take-away sweets and fast food (0.5, 1.0, and 2.5 km) were susceptible to depression. These associations were modified by undergraduates' socioeconomic attributes (e.g., grade, *Hukou* status, and ethnicity) and varied according to geographical scales and exposure metrics.

**Conclusion:**

To deliver effective environmental interventions to curb the prevalence of depression among undergraduate students, further planning policies should focus on the careful conception of the campus-based environment, especially regarding different spatial scales.

## Background

The university period is a critical stage of emerging adulthood (1) during which time individuals are often faced with increasing expectations from their families and society. When there is a perceived failure to meet these expectations, the onset of common mental disorders, such as depression, and risky behaviors, such as suicide, may arise (2, 3). Depression is prevalent among university students in many regions of the world (2, 4, 5) and affects the quality of life, relationships, academic attachment, and work opportunities of many students (6). Many studies reported over 30% of university students suffer from depression (2, 5), which is much higher than global and national levels in China (6, 7). The latest report on national mental health development in China (2019–2020) reported that roughly 18.5% university students were depressed with depressive scores (assessed by the Center for Epidemiological Studies Depression Scale, CES-D) ranging from 10 to 17, and 4.2% of other university students are at high risk of depression with CES-D scores over 17 (8). To respond to the high prevalence and well-documented negative effects of depression in university students, depression screening and prevention have attracted the attention of policy-makers.

The high prevalence rate of depression could be attributed to biological characteristics and external environmental factors, including social, natural, and built components of the environment (2, 9). Unlike adolescents, biologically, most university students are at a point in emerging adulthood where they “have reached physical and sexual maturity, and are highly diverse in their educational and occupational combinations and trajectories, p. 569” (1). In contrast with adults, the majority of university students are not yet in stable long-term romantic or career commitments (1). Consequently, most university students often experience stress, anxiety, and depression (2, 5, 10–12). In addition, most undergraduates in China live and learn on campuses with unique environments and corresponding management modes (12). To mitigate students' living expenditures and facilitate student management, universities typically offer or compel students to live in the low-cost dormitories within or near the campus (13). As a result, undergraduates have hardly any freedom to choose their residences (14). In addition, students perform most of their daily routines (e.g., learning, living, eating, etc.) within or surrounding these campuses (12). Given these factors, there are grounds to explore the implications of campus environment on the mental status of students.

Numerous studies have associated geriatric, adolescent, and pre-natal depression with exposure to natural and built environments (NBEs), especially in regards to the residential and working neighborhoods that are central to people's daily activities (15–20). However, little is known regarding depression in undergraduates in this context. The theoretical and empirical evidence points to the potential of NBEs to reshape depression-related behaviors, including physical activities, social contacts, etc., but their effects on depression itself remains mixed when it comes to different environmental variables and populations (21). Natural spaces, especially green spaces show potential to reduce depressive moods (19, 22–24) and improve mental health (25, 26) through stress relief (11), physical activity (PA) (27, 28), and social cohesion (24, 29). Most existing findings relevant for human benefits in relation to depression have been associated with generalized greenness (commonly captured by the normalized difference vegetation index [NDVI] or by overall greenery coverage) (19, 24, 26, 30), and very few studies have focused on specific types of green spaces (31–33). As reported, the associations between depression/depression-related behaviors and greens spaces differ depending on the type of green space concerned (34, 35). For instance, Giles-Corti et al. suggested the positive effects of flat grassy areas on facilitating social and physical recreation, but not walking, in older adults (34), whereas Holtan et al. associated social capital increase with the presence of tree canopies but not the presence of parks and grass (35). Built environments are broadly defined as human-made facilities and infrastructures for supporting human activities (25). Based on Ewing and Cervero's “5D” model (36), over 100 objective measures of built environments can be used to understand the relationship between built environment and mental health (37). Among these measures, food facilities and road/street environments are closely related to students' daily activities. Road/street environments are more likely to be related to physical activity (38), and food facilities are more likely to change dietary patterns (39), both of which could affect depression (40–42).

Although there is growing scientific recognition of the effect of NBEs on depression and depression-related behaviors (15, 19, 23, 38), there are still some limitations to investigating the relationships between campus environments and depression in undergraduates. First, there is limited relevant research regarding undergraduates who live in distinctive environments and take part in unique daily activities (12, 13). Second, few studies have been performed to examine how depression is correlated with different types of green spaces, which is meaningful to urban planning and decision-making (30, 31). In addition, residential self-selection bias (43–45) and the uncertain geographic context problem (46, 47) both affect research in this field. Residential self-selection implies that participants are likely to choose their neighborhood according to their lifestyle and personal preferences, so those who are healthy, or want to be healthy, may choose to live in a neighborhood with better environmental quality (e.g., places with greener spaces and better walkability) (44, 45). This kind of bias can affect the relationship between health outcomes and exposure to such environments (44, 45), but has rarely been addressed in previous studies (16, 24, 26). Investigating a subgroup population with little freedom to choose their residential location is recommended as an effective solution to mitigate this type of bias because of the high cost for longitudinal and (quasi) experimental research and the difficulties in distinguishing preferences and attitudes (14, 45). To relieve cost and ensure the safety of students, Chinese universities offer dormitories within or near the campus at which residence is compulsory (13), thereby constraining their choices of where to live and restricting most of their routine activities (i.e., learning, playing sports, eating, and living) within the campus environment (12). The uncertainty of geographic context is another undeniable problem relevant to environmental health (46, 47). The measures of exposure to NBEs varies with the definition of neighborhood (i.e., buffer sizes, buffer shapes, etc.), causing mixed findings regarding the relationship between NBEs and depression (38, 48). Empirically, multiple-scale environmental measures have been captured to solve this issue (38, 48).

Last but not least, individual socio-economic status (SES), such as gender, ethnicity, age, education status, and economic condition, have been reported to moderate the association between NBEs and depression (18, 19, 48), and the moderating roles are reported to differ in varying populations. For instance, green spaces are protective against depression among low-educated pregnant women, but this moderating role is not significant for their children's mental wellbeing (48, 49). Therefore, it is necessary to examine the moderating role of individual attributes on this association among undergraduates. Further, this is conducive to identifying subgroups for whom interventions to change the NBEs might be the most effective.

**Figure 1:**
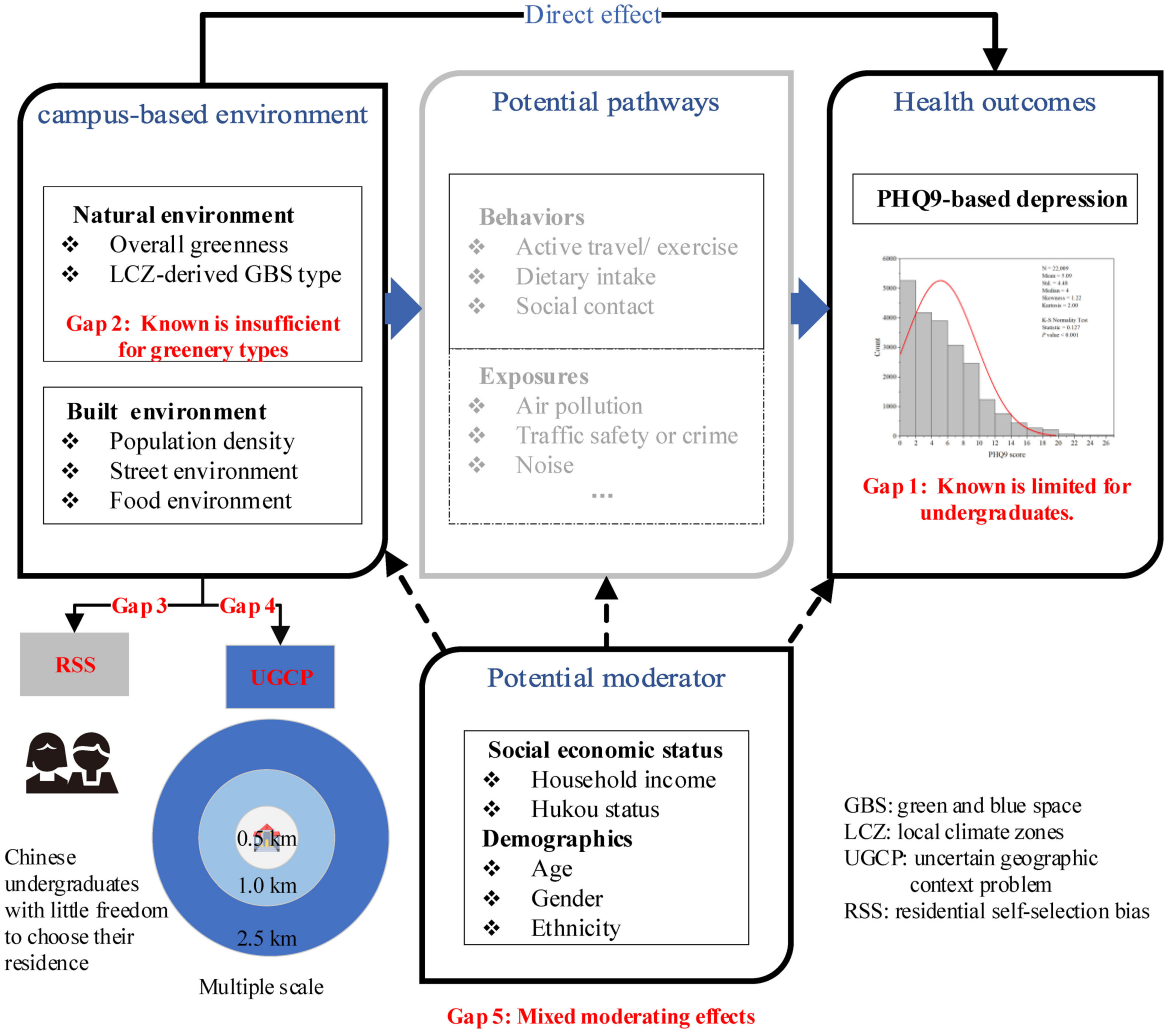
Conceptual framework illustrating how campus-based environmental factors affect depression.

Given the above gaps in the literature (25, 30) and the daily routines of undergraduates in China (see Supplementary Figure 1), a conceptual framework (Figure 1) was proposed to illustrate the underlying mechanisms for how the campus-based environments affect depression. Subsequently, a nationwide representative sample of 22,009 undergraduates from 89 campuses across China was used to examine the associations between depression in undergraduates and campus-centered natural-built environments at multiple spatial scales (i.e., 0.5, 1, and 2.5 km)^1^. The moderating role of individual socioeconomic attributes was examined after RSS was disentangled. This study contributes to the literature in five aspects: First, it is the original attempt to relate the incidence of depression in undergraduates to NBEs within and surrounding campus environments where undergraduates' living and working conditions are combined (after controlling for RSS bias). Second, this study enhances our understanding of how different types of green spaces affect depression. Third, the uncertainty of geographic contexts is compared at multiple geographical scales (0.5, 1, and 2.5 km). Fourth, the association between NBEs and depression are not confounded by residential self-selection bias in this study. Finally, the individual-based moderating role of environment-depression associations among undergraduates is clarified through interaction analysis.

## Methods and Data

### Data

**Figure 2:**
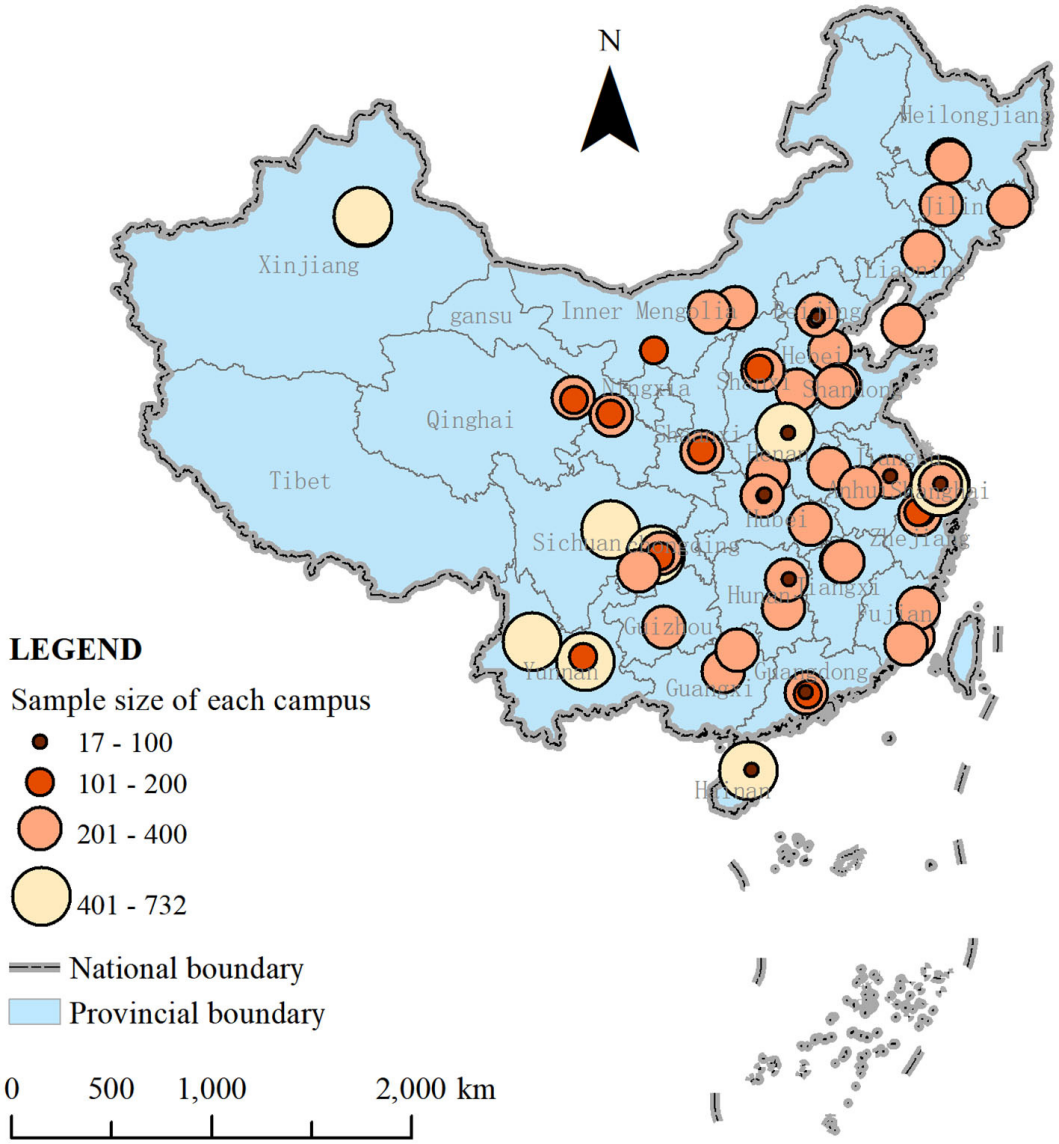
Spatial distribution and sample size of the surveyed campuses.

In this study, individual information was derived from a nationwide university-based survey on Chinese undergraduates conducted in 2018 (Ethics No. 2018-L-25). Using a stratified, multiple-stage cluster sample design, the research team generated a representative sample of 23,488 undergraduates from 90 campuses in 29 provincial units (Figure 2) after excluding 192 participants owing to missing data.

**Figure 3:**
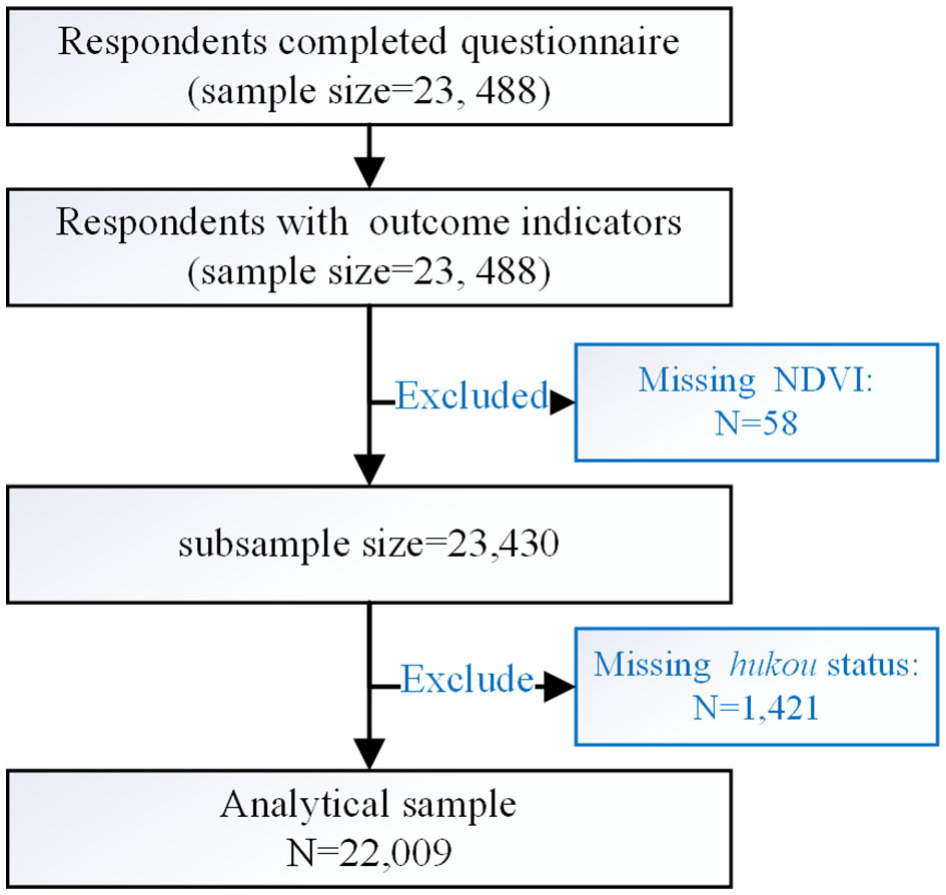
Flowchart for participant selection.

A structured questionnaire—designed by a multidisciplinary expert panel consisting of experienced epidemiologists and healthcare professionals—was used to collect data including socioeconomic characteristics, patterns of trips for routine activities, and health status. A set of natural and built environmental measurements was extracted by the GIS method according to the geocoded address of the campuses. According to prior studies (37, 50) and the transportation distances of participants (Supplementary Figure 1), functional neighborhoods were created for three distances of 0.5, 1, and 2.5 km. After excluding respondents with missing NDVI (*n* = 58) and *Hukou* status (*n* = 1,421), the final analytical sample comprised 22,009 respondents from 89 campuses (Figure 3).

### Outcome

Depression severity was measured by the nine-item Patient Health Questionnaire (PHQ9, Supplementary Table 1) (51), one of the most widely used tools to measure depression severity for the previous 2 weeks in non-clinical populations (19, 52). This scale has been verified in the Chinese general population (53). Each item is scored from 0 to 3, and the sum scores could range from 0 to 27 (Supplementary Figure 2), with higher scores indicating more severe depression. The sum scores of PHQ9 < 5 is usually recognized as minimal or no depression (2, 51).

### Environmental Exposure

#### Natural Environments

Empirical evidence on impact of NDVI on depression is conclusive (19, 20, 54); however, which type of green space has a greater effect on depression is not yet well-understood (30, 31). In this paper, we focus on the benefits of NDVI and five types of urban natural features (i.e., dense tree, scattered trees, bush/scrubs, low plants, and water) derived from local climate zone maps, for the reduction of depression. The NDVI index was calculated by the spectral reflectance measurements acquired in the near-infrared regions (760–900 nm) and visible red region (630–690 nm) retrieved from the Sentinal-2 satellite data with a high spatial resolution (10 × 10 m) in 2018 (55). The values of the unit-less index range from−1 to 1, with higher values indicating a higher level of green vegetation. The coverage ratio of each landscape type was derived from a 30 m Landsat 8 level 1 image of land cover according to the 2018 local climate zone map provided by the Hong Kong University through the mapping on the World Urban Database and Access Portal Tools (56). Local Climate Zones (LCZ) were developed as a classification system consisting of seven types of land cover, five of which can be described as green and blue spaces (GBS), including dense trees (LCZ_A), scattered trees (LCZ_B), bush, scrub (LCZ_C), low plants (LCZ_D), and water (LCZ_G) (57, 58).

#### Built Environments

The built environments were measured *via* three main categories depending on their influence on undergraduates' routine activities. As depression has been associated with urbanicity, population density was captured as a proxy of urbanicity, based on the assumption that urbanicity is correlated with population density (16). This was measured as the number of people per square kilometer, as reported by WorldPop in 2018, with a resolution of 100 × 100 m (14). Streets are an internal component of open spaces in cities and have attracted attention from scholars because of their close relation to transportation, especially modes of active travel (38, 59), which are protective against low levels of depression (60, 61). Three measures including street intersection density, road network density, and bus stop density were captured to characterize campus-based street environments. However, road network density and bus stop density were excluded because of collinearity. The number of street intersections (three-way or more), bus stops, and the lengths of road networks in the defined buffer were obtained from Open Street Map in 2018. The presence of diverse food outlets has previously been reported to be correlated with people's dietary patterns (39, 62). Unhealthy dietary patterns such as the consumption of sweets and high-fat food are associated with increased risk for depression (40, 41). Accordingly, food environments were measured based on the number of fast-food restaurants and take-away sweet shops (e.g., bakery shops, ice cream shops, and dessert house) per square kilometer. The count of each food outlet was retrieved from points of interest (POIs) data from the Gaode map (one of the largest map providers in China) in 2018.

### SES Indicators and Other Covariates

Undergraduates who are older, female, and of lower socioeconomic status are more likely to report depression (2). The prevalence of depression also varies across rural and urban areas (7), and ethnic groups (48). Following prior studies in China (16, 24), the individual SES attributes of age, gender, ethnicity, *Hukou* status, and household income, were viewed as potential moderators of the association between environment and depression.

In addition, other individual and campus-based covariates were controlled including body mass index (BMI) and level of physical activity required for transportation (T_PA_) at the individual and urbanization level, and geographical variation and university type at the campus-based level. The BMI index was calculated by dividing weight in kilograms by the square of height in meters by *in situ* measurement, while T_PA_ was defined as total time spent weekly on active travel (walking and cycling). T_PA_ was generated according to respondents' answers to questions about the frequency (F_i_), distance (D_i_), and average velocity of travel modes (V_i_) for trips to seven categories of daily activities—learning, exercising, shopping, visiting friends, recreation, visiting the doctor, and working or doing internships. The V_i_ value*s* were assumed to be 15, 5, and 0 km/h for cycling, walking, and other transport modes, respectively (63). The relative deviation of T_PA_ was expressed as tertiles.

According to campus locality, the urbanization level was operationally defined as urban and suburban (14), and geographical variations were operationally defined as eastern, central, and western zones (64). School type was determined according to the Chinese university ranking system (high and general) as established by the National Ministry of Education. As reported, university students from higher level universities are expected to experience high levels of stress because of intense competition and pressure, which may in turn increase depression (11).

### Statistical Analysis

Although the PHQ9 score was measured *via* arbitrary scales and not truly continuous (Supplementary Figure 2), the outcome was treated as continuous because there were more than five distinct values (0–27) for depression (65). Multivariate linear regressions were performed to explore influencing factors of depression at multiple scales (Model 1) for the low infra-class correlation (ICC = 0.03<0.06) in the null model and the robustness of linear regression modeling against moderate violations of the normal distribution assumption (65). Subsequently, multiplicative interaction terms were constructed to examine possible moderating roles of individual socioeconomic attributes on the associations between the frequency and severity of depression in undergraduates and exposure to natural and built environments within and surrounding campuses (Model 2). Variance inflation factor (VIF) values below 4 (Supplementary Table 2) were used to identify and control for multicollinearity among the independent variables (45), which excluded the road network and bus stop densities. To further avoid multicollinearities, the interaction term was generated by centered variables in interaction models. The Akaike Information Criterion (AIC) was introduced to compare the quality of these models; low AIC scores indicate a better model fit (16). The unstandardized coefficients (Coef.) and standardized error (SE) were reported for regression results. All statistical analyses were performed using IBM SPSS Statistics 26.0 and STATA 16.1.

## Results

### Descriptive Statistics of Samples

As seen in Table 1, the PHQ9 score ranged from 0 to 27 in undergraduates, with a median of 4, standard deviation of 4.48 (Table 1), and 47.7% were reported with depression (PHQ9 ≥ 5) of different degree (see Supplementary Figure 2). Although the average level of depressive score in undergraduates are reported with no depression (PHQ9 ≤ 4) (51), the relevant prevalence of depression is higher than previously reported rates. As reviewed, overall 30.6 and 24.4% of university students have experienced depressive symptoms worldwide (2) and in low-/middle- income countries (66). Additionally, the reported prevalence of depression was 14.9 and 24.3% among university students the United State and Malaysians (67, 68).

**Table 1:** Descriptive statistics of 22,009 participants.

**Characteristics**	***N* (proportion, %)**	**Mean (SD)**	**Median (IQR)**
**Outcome**			
PHQ9		5.09 (4.48)	4.00 (6.00)
**Individual socioeconomic attributes**			
Age		20.01 (1.75)	20 (2)
Gender: Male (ref. Female)	9,779 (44.43)		
Ethnicity: Minorities (ref. Chinese-Han)	2,955 (13.42)		
*Hukou* status before college enrollment: Rural (ref: urban)	8,683 (39.45)		
Annual household income: High (ref.low)	6,879 (31.26)		
Duration of exposure: Freshmen (ref. senior)	5,908 (26.84)		
**Covariates**			
BMI (kg/m^2^)		20.56 (2.73)	20.08 (3.26)
T_PA_ (hour/week): (ref: active)			
Inactive	7,362 (33.45)		
Moderate	7,354 (33.41)		

*BMI, body mass index; SD, standard deviation; IQR, interquartile range*.

*T_PA_, Time spent weekly on physical activity for non-motorized mode. According to tertiles of T_PA_, this indicator were categorized into: inactive (≤ 1.0 h/week), moderate (1.01–2.35 h/week) and active (≥2.36 h/week)*.

Of the 22,009 undergraduates, the majority were female, Chinese-Han, came from low-income families and urban places before enrolling in college, and had studied ≥ 1 year in the surveyed campus. The median T_AP_ and BMI were 1.58 h/week and 20.08 kg/m^2^, respectively.

As illustrated in Table 2, the average coverage ratios of dense trees, scattered trees, bush and scrub, and water, and population density increased with the buffer radius as it extended from 0.5 to 2.5 km. Density variables for street intersections, fast-food restaurants, and take-away sweets shops were the greatest with a 1.0 km radius, followed by a 2.5 km radius, and 0.5 km radius.

**Table 2:** Descriptive statistics of natural-built environmental features surrounding 89 campuses.

**Characteristics**	**Mean (25th−75th percentile)**		
	**0.5 km**	**1 km**	**2.5 km**
**Natural environments**			
NDVI	0.26 (0.20–0.31)	0.26 (0.21–0.31)	0.25 (0.20–0.29)
Dense trees	1.92 (0.00–0.79)	2.40 (0.00–1.25)	3.64 (0.07–2.90)
Scattered trees	0.69 (0.00–0.88)	0.85 (0.00–0.92)	1.14 (0.21–1.09)
Bush, scrub	2.51 (0.83–3.48)	2.51 (1.10–3.40)	2.65 (1.10–3.31)
Low plants	25.37 (9.26–34.75)	24.67 (11.83–34.22)	25.56 (14.76–33.79)
Water	11.67 (4.50–17.27)	12.73 (6.41–15.08)	14.64 (8.50–19.10)
**Built environments**			
Population density (10,000 population/m^2^)	0.85 (0.14–1.42)	0.98 (0.16–1.67)	1.02 (0.16–1.67)
Street intersection density (intersection/km^2^)	8.07 (3.82–11.46)	18.09 (8.28–23.25)	15.60 (7.80–18.34)
Fast food restaurant density (restaurant/km^2^)	19.49 (2.55–24.20)	19.63 (6.69–28.34)	14.53 (3.41–19.72)
Take-away sweet shops density (shop/km^2^)	7.90 (0.00–10.19)	8.75 (1.59–12.10)	6.68 (1.17–9.12)
**Campus-based covariates**			
Urbanization level: Suburban (ref. urban)^#^	44 (48.3%)	44 (48.3%)	44 (48.3%)
Geographic zone (ref. western)^#^			
Central	29 (32.6)	29 (32.6)	29 (32.6)
Eastern	33 (37.1)	33 (37.1)	33 (37.1)
University type: High (ref. general)^#^	26 (29.2)	26 (29.2)	26 (29.2)

*NDVI, normalized difference vegetation index*.

# *Denotes categorical variables*.

### Overall Associations Between Built Environment and Depression

Based on the unstandardized coefficients in Table 3, depression in undergraduates is significantly and negatively associated with scattered trees (0.5 km), water (0.5, 1.0, and 2.5 km), street intersection density (1.0 and 2.5 km), and population density (0.5 km). Inversely, undergraduates from campus with a higher density of outlets serving take-away sweets and fast foods (0.5, 1.0, and 2.5 km) are more likely to report higher depressive scores on the PHQ9. It is noteworthy that those impacts vary by different spatial scales.

**Table 3:** Results of multivariate linear associations between PHQ9-based depression and exposure to natural and built environments at multiple scales.

**Independent variables**	**Model 1a (0.5 km)**		**Model 1b (1 km)**		**Model 1c (2.5 km)**	
	**Coef. (SE)**	**β**	**Coef. (SE)**	**β**	**Coef. (SE)**	**β**
**Natural environments**						
NDVI^#^	−0.029 (0.040)	−0.007	0.034 (0.043)	0.008	0.070 (0.038)	0.018
Dense trees^#^	0.067 (0.051)	0.013	0.004 (0.031)	0.001	0.052 (0.041)	0.013
Scattered trees^#^	−0.201 (0.040)^**^	−0.039	−0.055 (0.034)	−0.014	0.020 (0.032)	0.005
Bush, scrub^#^	0.017 (0.034)	0.004	0.014 (0.034)	0.004	0.062 (0.033)	0.016
Low plants^#^	−0.016 (0.041)	−0.004	−0.047 (0.043)	−0.012	−0.047 (0.038)	−0.012
Water^#^	−0.105 (0.033)^**^	−0.026	−0.072 (0.030)^*^	−0.018	−0.115 (0.031)^**^	−0.028
**Built environments**						
Population density	−0.167 (0.049)^**^	−0.034	−0.013 (0.046)	−0.003	0.028 (0.052)	0.007
Intersection density	−0.034 (0.057)	−0.005	−0.135 (0.029)^**^	−0.045	−0.177 (0.046)^**^	−0.045
Fast-food restaurant density	0.005 (0.001)^**^	0.031	0.007 (0.002)^**^	0.026	0.009 (0.004)^*^	0.026
Take-away sweet shops density	0.014 (0.003)^**^	0.032	0.010 (0.003)^**^	0.022	0.015 (0.004)^**^	0.024
**Individual socioeconomic attributes**					
Age	−0.019 (0.021)	−0.007	−0.016 (0.021)	−0.006	−0.014 (0.021)	−0.005
Gender (ref. female)	−0.300 (0.064)^**^	−0.033	−0.331 (0.064)^**^	−0.037	−0.333 (0.063)^**^	−0.037
Duration of exposure (ref. freshmen)	0.683 (0.081)^**^	0.068	0.657 (0.081)^**^	0.065	0.684 (0.081)^**^	0.068
*Hukou* status (ref. urban)	0.264 (0.065)^**^	0.029	0.298 (0.065)^**^	0.033	0.272 (0.065)^**^	0.030
Ethnicity (ref. Chinese-*Han*)	0.653 (0.092)^**^	0.050	0.671 (0.094)^**^	0.051	0.610 (0.093)^**^	0.046
Household income (ref. high)	0.298 (0.069)^**^	0.031	0.292 (0.069)^**^	0.030	0.288 (0.070)^**^	0.030
**Covariates**						
BMI (kg/m^2^)	−0.008 (0.012)	−0.005	−0.009 (0.012)	−0.005	−0.009 (0.012)	−0.006
PA: inactive (ref. active)	0.171 (0.074)^*^	0.018	0.157 (0.074)^*^	0.017	0.152 (0.074)^*^	0.016
moderate (ref. active)	0.143 (0.074)	0.015	0.137 (0.074)	0.014	0.137 (0.074)	0.014
Urbanization: urban (ref. suburban)	0.188 (0.076)^*^	0.019	0.151 (0.076)^*^	0.015	0.147 (0.079)	0.015
Geographical zone: (ref: western)						
Central (ref. western)	0.143 (0.083)	0.014	0.157 (0.084)	0.016	0.115 (0.082)	0.012
Eastern (ref: western)	0.273 (0.081)^**^	0.029	0.361 (0.085)^**^	0.038	0.442 (0.085)^**^	0.045
University type: High (ref. general)	0.262 (0.078)^**^	0.027	0.110 (0.079)	0.011	0.114 (0.082)	0.012
**Goodness of model fit:**						
Sample size	22,009		22,009		22,009	
AIC score	125,154		128,175.5		128,333.6	

*Data reported with unstandardized regression coefficient (standardized error, SE); β is standardized regression coefficient*.

AIC, Akaike Information Criterion.

# *Effect estimates are reported per interquartile range increase*.

** *p < 0.01*;

* *p < 0.05*.

The standardized coefficients (β) in Table 3 suggest that there are the highest negative associations between depression and scattered trees and population density. By contrast, the negative relations between depression and water decreased, with a buffer zone of 2.5 km showing the greatest association and a buffer zone of 1.0 km showing the weakest association. Additionally, the negative relationships between depression and street intersection density showed no difference across buffer distances. Unlike aforementioned negative associations, the positive correlation between depression and food outlets for take-away sweets and fast foods was greatest with a buffer zone of 0.5 km and weakest with a buffer zone of 1.0 km.

### Other Determinants for Depression

In terms of individual covariates (Model 1 a – c), they played significant roles as expected. Specifically, female, senior, and ethnic minority undergraduates reported higher depressive scores. Urban *hukou* status before enrollment and high family income were positively and significantly related to higher depressive scores. Additionally, physical inactivity was positively correlated with depressive scores. Regarding campus-based covariates (Model 1 a – c), undergraduates from campuses located in eastern region (0.5, 1.0, and 2.5 km) and urban fields (0.5 km) were more likely to become depressed. In addition, undergraduates studying in high-level universities were more likely to suffer from severe depression (0.5 and 1.0 km).

### Moderating Effects of Socioeconomic Factors

Table 4 and Supplementary Tables 3–5 reveal the moderating role of socioeconomic attributes on the association between environment and depression. The negative association between depression and water within a 0.5 km buffer zone is greater in undergraduates from urban cities than rural areas, but the association between depression and number of street intersections was higher in senior (1.0 and 2.5 km), ethnic (2.5 km), and urban-origin (1.0 and 2.5 km) undergraduates than their reference groups. The positive relationship between depression and take-away sweet shop density was stronger in Chinese-*Han* (0.5 km) and rural (2.5 km) undergraduates. There was no moderating role found for other associations.

**Table 4:** Results of multivariate linear associations between depression in undergraduates and exposure to natural-built environments at multiple scales and with interaction effects of socioeconomic attributes.

**Independent variables**	**Potential moderators**				
	**(–) Gender**	**(+) Duration of exposure**	**(+) *Hukou* status**	**(+) Ethnicity**	**(+) Household income**
	**(ref. female)**	**(ref. freshmen)**	**(ref. urban)**	**(ref. Chinese-*Han*)**	**(ref. high)**
**Model 2a (0.5 km)**					
(-) Scattered trees^#^	−0.113 (0.069)	−0.032 (0.078)	0.044 (0.074)	−0.079 (0.103)	0.085 (0.077)
(-) Water^#^	−0.029 (0.055)	0.043 (0.064)	−0.192 (0.059)^**^	0.004 (0.085)	0.103 (0.062)
(-) Population density	−0.015 (0.067)	−0.151 (0.080)	−0.027 (0.075)	0.095 (0.110)	0.010 (0.074)
(+) Fast-food restaurant density	−0.0002 (0.002)	0.001 (0.003)	0.002 (0.002)	0.005 (0.004)	0.003 (0.003)
(+) Take-away sweet shops density	0.011 (0.003)	−0.004 (0.006)	0.002 (0.006)	−0.017 (0.008)^*^	0.001 (0.007)
**Model 2b (1 km)**					
(-) Water	0.011 (0.056)	0.057 (0.063)	−0.083 (0.059)	0.095 (0.087)	0.056 (0.062)
(-) Street intersection density	−0.016 (0.041)	−0.161 (0.049)^**^	0.067 (0.049)	0.194 (0.069)^**^	−0.065 (0.044)
(+) Fast-food restaurant density	−0.002 (0.004)	−0.005 (0.004)	−0.001 (0.004)	−0.005 (0.006)	−0.001 (0.004)
(+) Take-away sweet shops density	0.006 (0.006)	−0.005 (0.007)	0.008 (0.007)	−0.007 (0.009)	−0.009 (0.007)
**Model 2c (2.5 km)**					
(-) Water	−0.031 (0.056)	−0.099 (0.063)	−0.080 (0.059)	0.128 (0.085)	−0.069 (0.063)
(-) Street intersection density	−0.048 (0.054)	−0.256 (0.068)^**^	0.138 (0.065)^*^	0.217 (0.091)^*^	−0.045 (0.059)
(+) Fast-food restaurant density	−0.001 (0.005)	−0.017 (0.006)^**^	−0.001 (0.005)	−0.001 (0.008)	−0.005 (0.005)
(+) Take-away sweet shop density	0.006 (0.008)	−0.016 (0.009)	0.018 (0.009)^*^	0.002 (0.013)	−0.008 (0.009)

*Data reported with unstandardized regression coefficient (standardized error)*.

# *Effect estimates of exposure to natural environments are reported per interquartile range increase*;

** *p < 0.01*;

* *p < 0.05*.

The relationships of explanatory variables and potential moderators with PHQ9 score were expressed with “+” and “-” in the in brackets.

Effect estimates of scattered trees and water coverage are reported per interquartile range increase.

*Socioeconomic factors and covariates in Model 1 were adjusted in Model 2*.

## Discussion

### Specific Types of Natural Spaces, Not NDVI, Protect Against Depression

In contrast to the expectation that more NDVI mitigates depression (19, 48), the association failed to reach statistical significance in this study, which is consistent with some other previous findings (20, 54). Similar to prior studies (33, 69), however, more scattered trees within campuses helped to protect against depression. The spatial variation of protective effects of scattered trees on depression may be attributed to the fact that Chinese undergraduates conduct their daily activities within the campus. In contrast to some of the available evidence (20, 70), the prevention of depression was positively associated with an increase in coverage rate of water.

### The Effect of the Built Environment on Depression Depends on Features and Spatial Scale

The built environment affects health through either modifying environmental exposure or reshaping behaviors (25). Some previous studies hold that population density mitigates depression as there are more opportunities to contact neighbors or friends (71) and to enjoy medical resources (72), thereby promoting health (16). However, this significant association was only observed within 0.5 km buffers (Table 3). One possible explanation is that the majority of Chinese undergraduates conduct their routine activities within and near campus, as they are required to complete most of their studying on campus and to live in dormitories within the campus or in the surrounding areas (12, 13). Another explanation for the inconclusive association is that most undergraduates can obtain good and affordable medical resources in school hospitals. Improvements to street connectivity create a more walkable neighborhood (73, 74) and encourage active travel such as walking and cycling (38), thereby preventing depression (75). Accordingly, more street intersections surrounding the campus can reduce depression by facilitating PA for transport and reducing the hazardous environmental exposure generated by vehicle transportation. Street intersection density is a commonly used measure of street connectivity that provides direct and safe pathways for active trips (38). Similar to a prior study (76), unhealthy food facilities serving fast foods and take-away sweets might shape a dietary pattern of sweets and high-fat foods (39), both of which are risk factors for depression (40–42).

### Moderating Role of Socioeconomic Attributes

A better understanding of the moderating role that individual socioeconomic attributes have on the connections between NBEs and depression is conducive to identifying subgroups for whom interventions to improve NBEs might be the most effective. Increasingly, studies have focused on the moderating role of socioeconomic status, race/ethnicity, gender, age, and urbanicity on the relationships between depression and environment (18, 24, 48), but conclusions vary significantly in space, over time, and across population subgroups. Unlike previous studies on the moderating role of the health benefits of green spaces (48), we found no differences in the associations between scattered trees and depression based on household income and other potential moderators. In contrast, undergraduates from urban areas were generally more likely to benefit more from water (0.5 km), and subsequently experience lower levels of depression.

The presence of more street intersections appears to relate to depression more significantly in senior, urban, and ethnic minority students. This may be attributed to discrepancies in mobility and interaction among these students. Senior undergraduates generally have more opportunities to interact with this transportation infrastructure because of working or doing internships. Undergraduates originating from urban areas may visit destinations (e.g., shopping malls) far away from campus more frequently in their leisure time compared to those from rural areas (14). Additionally, a well-connected campus enables ethnic undergraduates more choices for non-motorized transportation (59). Consumption habits might be a possible explanation for the higher positive association between depression and take-way sweet facilities among rural undergraduates. Compared to students from urban areas who are used to more food choices, rural undergraduates' diet patterns could be more greatly affected by surrounding food facilities.

### Implications for Public Health and Urban Planning

Depression is a leading cause for global disability and has been a cause of worldwide concern. Although there is huge variation across studies, the prevalence of depressive disorders in university students is considerably higher than rates reported in the general populations (2, 5–7). On average, 47.7% of Chinese undergraduates experienced depressive symptoms, which is much higher than the overall rates among university students in the globe (30.6%) and in the low-/middle- income countries (24.4%), as well as the 14.9% in the United State (2, 66, 67). Our findings regarding biological and environmental risk and protective factors for depression provide foundations for the prevention of not only depressive disorders, but also other complications, and in turn the reduction of disease and financial burdens.

In response to the high and increasing prevalence of depression in undergraduates, this study has significant implication for urban planning and public health promotion, for both China or other low- and middle-income countries. Our findings confirmed that scattered trees (but not overall greenery) and water within a 0.5 km buffer zone help alleviate depressive symptoms, as previously suggested in literature from other regions. First, campus designers and mangers should consider the effects of specific types of land-cover on depressed mood when seeking to improve or create new campuses. Second, urban designers and planners should include comprehensive plans for nearby areas, because undergraduates often are also exposed to the areas surrounding campuses. For example, better street connectivity and healthier food environments should be considered. Third, socioeconomic differences in the association between campuses and undergraduates show the importance of the dissemination of information regarding and promotion of health lifestyles by campus administrators.

### Limitations

Despite our study's contributions to the literature, it also has several limitations. First, it follows a cross-sectional design, which precludes identifying causality between depression and exposure to NBEs. Second, static rather than dynamic or time-series exposure to NBEs was used to identify their effect on depression, although the duration of exposure was operationalized by the grade. Third, data sources on depression and individual covariates could be affected by recall bias, although face-to-face interviews could mitigate this bias to a certain extent. Fourth, since participants' medical and family histories of depression and other emotional disorders were inaccessible, the analyses could not be adjusted for these factors. Finally, measures of overall greenness and natural-land covers including dense trees, scattered trees, bush and scrubs, low plants, and water are operationally defined and characterized by different datasets under different resolutions, there thus might be some misclassification of exposure.

## Conclusion

This study provides the first nationwide empirical evidence regarding the association between the severity of depression in Chinese undergraduates and the natural and built environmental characteristics within and surrounding their campus environments. After disentangling RSS, we found that natural landscapes and built environments can influence depression, but their effects varied by geographic spatial scales. Scattered trees and water had a protective effect but overall greenness or other landscape types had no association with depression. A well-connected campus buffers against depression. Conversely, more access to food outlets serving fast food and take-away sweets is related to higher levels of depression. Apart from gender and household income, the other individual socioeconomic attributes including ethnicity, *Hukou* status, and duration of exposure, were found to modify the associations between depression and campus environments, although the moderating roles varied across spatial scales and exposure metrics. The information from this nationwide study has implications that can be used to guide city planning for the improvement of campus environments by the management of natural and physical settings within and surrounding campuses. Further studies, not limited to depression, with prospective cohorts or quasi-experimental designs, are needed to clarify how natural and physical settings surrounding campuses affect the health of college students.

## Data Availability Statement

The original contributions presented in the study are included in the article/Supplementary Materials, further inquiries can be directed to the corresponding authors.

## Ethics Statement

The studies involving human participants were reviewed and approved by First Affiliated Hospital of Kunming Medical University (Ethical number: 2018-L-25). Written informed consent to participate in this study was provided by the participants' legal guardian/next of kin.

## Author Contributions

HY and XC conceived the study. XC conducted the data analysis. HY prepared the draft of the manuscript. MD revised the manuscript. JC collected information on the campus-based environmental measurements. All authors provided critical feedback on all versions of the manuscript. All authors have read and approved the final manuscript.

## Funding

This work was supported by the Strategic Priority Research Program (A) of the Chinese Academy of Sciences (Project No. XDA19040402) and the Shanghai Pujiang Program (2019PJC034).

## Conflict of Interest

The authors declare that the research was conducted in the absence of any commercial or financial relationships that could be construed as a potential conflict of interest.

## Publisher's Note

All claims expressed in this article are solely those of the authors and do not necessarily represent those of their affiliated organizations, or those of the publisher, the editors and the reviewers. Any product that may be evaluated in this article, or claim that may be made by its manufacturer, is not guaranteed or endorsed by the publisher.

## Footnotes

^1^A radius of 1.0 km was commonly used to define geographical buffer zone in prior studies. A radius of 0.5 km buffer zone was used to characterize the environments within campus and a radius of 2.5 km was used to characterize the environmental features (1.0–3.0 km) where considerable undergraduates were transported.
