# Supplementary material for: Association Between Natural/Built Campus Environment and Depression Among Chinese Undergraduates: Multiscale Evidence for the Moderating Role of Socioeconomic Factors After Controlling for Residential Self-Selection
Source: Front Public Health. 2022 Apr 7;10:844541. doi: 10.3389/fpubh.2022.844541 (PMC9037627; doi:10.3389/fpubh.2022.844541)
Supplement: Supplementary file 1 [file Table_1.docx]

Supplementary Material

Association between the natural-built environment and depression among Chinese undergraduates: Multiscale evidence for the moderating role of socioeconomic factors after controlling for residential self-selection

Haoran Yang ^a^ [(](mailto:(haoranyang0119@126.com)[haoranyang0119@126.com](mailto:(haoranyang0119@126.com)), Xaingfen Cui ^b *^ ([cui1987rainny@163.com](mailto:cui1987rainny@163.com)),

Martin Dijst ^c^ ([Martin.Dijst@liser.lu](mailto:Martin.Dijst@liser.lu)), Senlin Tian ^b^ ([tiansenlin@outlook.com](mailto:tiansenlin@outlook.com)),

Jie Chen ^d, *^ ([chenjie@kust.edu.cn](mailto:chenjie@kust.edu.cn)), Jianhong Huang^b^ ([huangjianhong78@163.com](mailto:huangjianhong78@163.com))

Haoran Yang ^1^, Xiangfen Cui ^2^ *, Martin Dijst ^3^, Senlin Tian ^2^, Jie Chen ^4, *^, Jianhong Huang^2^

^1^The Centre for Modern Chinese City Studies & Research Center for China Administrative Division & Future City Lab, East China Normal University, Shanghai, China

^2^Faculty of Environmental Science and Engineering, Kunming University of Science and Technology, Kunming, China

^3^Department of Urban Development and Mobility, Luxembourg Institute of Socio-Economic Research (LISER), Esch-sur-Alzette, Luxembourg

^4^Faculty of Land Resource Engineering, Kunming University of Science and Technology, Kunming, China

*Corresponding author: Faculty of Environmental Science and Engineering, Kunming University of Science and Technology; E-mail: [cui1987rainny@163.com](mailto:cui1987rainny@163.com); Faculty of Land Resource Engineering, Kunming University of Science and Technology; E-mail: [chenjie@kust.edu.cn](mailto:chenjie@kust.edu.cn)

# Supplementary Tables

Supplementary Table 1 Nine-symptom checklist of nine-item Patient Health Questionnaire (PHQ-9)

| **Over the last 2 weeks, how often have you bothered by any of the following problems?** | | **Not at all** | **Several days** | **More than half the days** | **Nearly every day** |
| --- | --- | --- | --- | --- | --- |
| Q1 | Little interest of pleasure in doing things | 0 | 1 | 2 | 3 |
| Q2 | Feeling down, depressed, or hopeless | 0 | 1 | 2 | 3 |
| Q3 | Trouble falling or staying asleep, or sleeping to much | 0 | 1 | 2 | 3 |
| Q4 | Feeling tired or having little energy | 0 | 1 | 2 | 3 |
| Q5 | Poor appetite or overeating | 0 | 1 | 2 | 3 |
| Q6 | Feeling bad about yourself-or that you are a failure or have let yourself or your family down | 0 | 1 | 2 | 3 |
| Q7 | Trouble concentrating on things, such as reading the newspaper or watching television | 0 | 1 | 2 | 3 |
| Q8 | Moving or speaking to slowly that other people could have noticed? Or the opposite-being so fidgety or restless that you have been moving around a lot more than usual | 0 | 1 | 2 | 3 |
| Q9 | Thoughts that you would be better off dead or hurting yourself in some way | 0 | 1 | 2 | 3 |
|  | | **Total score: ________________________** | | | |

Supplementary Table 2 Variance Inflation Factor and tolerance values for variables input to Model1~3

| **Independent variables** | **Model1a (0.5 km)** | | **Model1b (1 km)** | | **Model1c (2.5 km)** | |
| --- | --- | --- | --- | --- | --- | --- |
|  | **VIF** | **Tolerance** | **VIF** | **Tolerance** | **VIF** | **Tolerance** |
| **Natural environments** | | | | | | |
| NDVI | 2.08 | 0.48 | 2.42 | 0.41 | 2.16 | 0.46 |
| Dense trees | 2.23 | 0.45 | 1.64 | 0.61 | 2.23 | 0.45 |
| Scattered trees | 1.40 | 0.72 | 1.51 | 0.66 | 1.62 | 0.62 |
| Bush, scrub | 1.60 | 0.63 | 1.79 | 0.56 | 1.81 | 0.55 |
| Low plants | 2.29 | 0.44 | 2.48 | 0.40 | 2.22 | 0.45 |
| Water | 1.46 | 0.69 | 1.23 | 0.81 | 1.31 | 0.76 |
| **Built environments** | | | | | | |
| population density (10000 population/km^2^) | 2.15 | 0.46 | 2.50 | 0.40 | 3.65 | 0.27 |
| Intersection density (10 intersections/km^2^) | 1.82 | 0.55 | 2.04 | 0.49 | 2.99 | 0.33 |
| Distance to nearest bus stop(km) | 1.27 | 0.78 | 1.49 | 0.67 | 2.72 | 0.37 |
| Fast food restaurant density(restaurant/km^2^) | 1.34 | 0.75 | 1.15 | 0.87 | 1.12 | 0.89 |
| Take-away sweets shops (shop/km^2^) | 2.15 | 0.46 | 2.50 | 0.40 | 3.65 | 0.27 |
| **Individual socioeconomic attributes** | | | | | | |
| Age (*year, continuous*) | 1.44 | 0.69 | 1.46 | 0.69 | 1.46 | 0.69 |
| Gender (ref. female) | 1.12 | 0.90 | 1.11 | 0.90 | 1.11 | 0.90 |
| Duration of exposure (ref. freshmen) | 1.45 | 0.69 | 1.44 | 0.69 | 1.44 | 0.69 |
| Hukou status | 1.13 | 0.89 | 1.12 | 0.89 | 1.12 | 0.89 |
| Ethnicity (ref. minorities) | 1.11 | 0.90 | 1.13 | 0.88 | 1.13 | 0.89 |
| Annually household income (ref. low) | 1.15 | 0.87 | 1.15 | 0.87 | 1.16 | 0.86 |
| **Covariates** |  |  |  |  |  |  |
| BMI (kg/km^2^, continuous) | 1.10 | 0.91 | 1.10 | 0.91 | 1.10 | 0.91 |
| Active transport (ref. active): | 1.35 | 0.74 | 1.35 | 0.74 | 1.35 | 0.74 |
| Inactive | 1.35 | 0.74 | 1.35 | 0.74 | 1.35 | 0.74 |
| Moderate | 1.31 | 0.76 | 1.32 | 0.76 | 1.40 | 0.71 |
| Urbanization level (ref. suburban) | 1.56 | 0.64 | 1.57 | 0.64 | 1.50 | 0.67 |
| Geographic zone (ref. western) | 1.64 | 0.61 | 1.82 | 0.55 | 1.84 | 0.54 |
| Central | 1.43 | 0.70 | 1.47 | 0.68 | 1.56 | 0.64 |
| Eastern | 1.10 | 0.91 | 1.10 | 0.91 | 1.10 | 0.91 |
| University type (ref. general) | 1.35 | 0.74 | 1.35 | 0.74 | 1.35 | 0.74 |

Supplementary Table 3 Results of multivariate linear associations between undergraduates’ depression and exposure to NBEs within 0.5 km buffer zone with interaction effects of socioeconomics

| **Independent variables** | **β1 (S.E)** | **β2 (S.E)** | **β3 (S.E)** | **β4 (S.E)** | **β5 (S.E)** |
| --- | --- | --- | --- | --- | --- |
| **Natural environments** |  |  |  |  |  |
| NDVI^#^ | -0.036(0.004) | -0.037(0.040) | -0.030(0.040) | -0.031(0.040) | -0.030(0.040) |
| Dense trees^#^ | 0.072(0.051) | 0.064(0.051) | 0.066(0.051) | 0.067(0.051) | 0.059(0.051) |
| Scattered trees^#^ | -0.197(0.041) ** | -0.190(0.041) ** | -0.203(0.040) ** | -0.201(0.041) ** | -0.198(0.041) ** |
| Bush, scrub^#^ | 0.013(0.035) | 0.019(0.034) | 0.015(0.034) | 0.016(0.034) | 0.019(0.034) |
| Low plants^#^ | -0.007(0.042) | -0.009(0.042) | -0.012(0.042) | -0.014(0.042) | -0.016(0.041) |
| Water^#^ | -0.104(0.033) ** | -0.106(0.033) ** | -0.107(0.033) ** | -0.110(0.033) ** | -0.107(0.033) ** |
| **Built environments** |  |  |  |  |  |
| Population density (10000 population/km^2^) | -0.166(0.049) ** | -0.155(0.033) ** | -0.164(0.049) ** | -0.176(0.049) ** | -0.165(0.049) ** |
| Intersection density (10 intersections/km^2^) | -0.033(0.057) | -0.041(0.057) | -0.026(0.057) | -0.030(0.057) | -0.039(0.057) |
| Fast food restaurant density(restaurant/km^2^) | 0.005(0.001) ** | 0.005(0.001) ** | 0.005(0.001) ** | 0.005(0.001) ** | 0.005(0.001) ** |
| Take-away sweets shops (shop/km^2^) | 0.014(0.003) ** | 0.014(0.003) ** | 0.014(0.003) ** | 0.013(0.003) ** | 0.014(0.003) ** |
| **Interaction effects with significant NBEs and individual attributes** |  |  |  |  |  |
| c (Scattered trees ^#^) × c(gender) | -0.113(0.069) |  |  |  |  |
| c (Scattered trees^#^) × c (Duration of exposure) | -0.032(0.078) |  |  |  |  |
| c (Scattered trees^#^) × c (Hukou status) | 0.044(0.074) |  |  |  |  |
| c (Scattered trees^#^) × c(Ethnicity) | -0.079(0.103) |  |  |  |  |
| c (Scattered trees^#^) × c (Household income) | 0.085(0.077) |  |  |  |  |
| c (Water^#^) × c(gender) |  | -0.029(0.055) |  |  |  |
| c(Water^#^) × c (Duration of exposure) |  | 0.043(0.064) |  |  |  |
| c(Water^#^) × c (Hukou status) |  | -0.192(0.059) ** |  |  |  |
| c(Water) × c(Ethnicity) |  | 0.004(0.085) |  |  |  |
| c(Water) × c (Household income) |  | 0.103(0.062) |  |  |  |
| c (Population density) × c (gender) |  |  | -0.015(0.067) |  |  |
| c (Population density) × c (Duration of exposure) |  |  | -0.151(0.080) |  |  |
| c (Population density) × c (Hukou status) |  |  | -0.027(0.075) |  |  |
| c (Population density) × c (Ethnicity) |  |  | 0.095(0.110) |  |  |
| c (Population density) × c (Household income) |  |  | 0.010(0.074) |  |  |
| c (Fast food restaurant density) × c(gender) |  |  |  | -0.0002(0.002) |  |
| c (Fast food restaurant density) × c (Duration of exposure) |  |  |  | 0.001(0.003) |  |
| c (Fast food restaurant density) × c (Hukou status) |  |  |  | 0.002(0.002) |  |
| c (Fast food restaurant density) × c (Ethnicity) |  |  |  | 0.005(0.004) |  |
| c (Fast food restaurant density) × c (Household income) |  |  |  | 0.003(0.003) |  |
| c (Take-away sweets shops) × c (gender) |  |  |  |  | 0.011(0.006) |
| c (Take-away sweets shops) × c (Duration of exposure) |  |  |  |  | -0.004(0.006) |
| c (Take-away sweets shops) × c (Hukou status) |  |  |  |  | 0.002(0.006) |
| c (Take-away sweets shops) × c (Ethnicity) |  |  |  |  | -0.017(0.008) * |
| c (Take-away sweets shops) × c (Household income) |  |  |  |  | 0.001 (0.007) |
| **Goodness of model fitting:** |  |  |  |  |  |
| Sample size | 22,009 | 22,009 | 22,009 | 22,009 | 22,009 |
| AIC | 128,158.7 | 128,152.0 | 128,159.6 | 128,159.7 | 128,155.5 |

NBEs: natural and built environments;

The variable started by “c” represented the variable were centered;

Data reported with coefficient (β) and standardized error (S.E.);

Effect estimates of NEs are reported per interquartile range increase; ** p<0.01; *p<0.05;

Models were adjusted by covariates including body mass index(kg/m^2^), tertiles of time spent weekly on physical activity for active travel, age and campus location.

Supplementary Table 4 Results of multivariate linear associations between undergraduates’ depression and exposure to NBEs within 1 km buffer zone with interaction effects of socioeconomics

| **Independent variables** | β1 (S.E) | β2 (S.E) | β2 (S.E) | β4 (S.E) |
| --- | --- | --- | --- | --- |
| **Natural environments** |  |  |  |  |
| NDVI^#^ | 0.033(0.043) | 0.035(0.043) | 0.032(0.043) | 0.029(0.043) |
| Dense trees^#^ | 0.003(0.031) | 0.005(0.031) | 0.005(0.031) | 0.007(0.031) |
| Scattered trees^#^ | -0.051(0.034) | -0.065(0.034) | -0.056(0.034) | -0.056(0.034) |
| Bush, scrub^#^ | 0.013(0.034) | 0.012(0.034) | 0.014(0.034) | 0.013(0.034) |
| Low plants^#^ | -0.043(0.043) | -0.047(0.043) | -0.046(0.043) | -0.044(0.043) |
| Water^#^ | -0.075 (0.030) * | -0.070(0.030) * | -0.072(0.030) * | -0.073(0.030) * |
| **Built environments** |  |  |  |  |
| Population density (10000 population/km^2^) | -0.009(0.046) | -0.017(0.046) | -0.011(0.046) | -0.009(0.046) |
| Intersection density (10 intersections/km^2^) | -0.135(0.029) ** | -0.133(0.030) ** | -0.132(0.029) | -0.138(0.029) ** |
| Fast food restaurant density(restaurant/km^2^) | 0.00 (0.022) ** | 0.007(0.002) ** | 0.007(0.002) | 0.007(0.002) ** |
| Take-away sweets shops (shop/km^2^) | 0.010(0.003) ** | 0.010(0.003) ** | 0.011(0.003) | 0.010(0.003) ** |
| **Interaction effects with significant NBEs and individual attributes** |  |  |  |  |
| c (Water^#^) × c(gender) | 0.011(0.056) |  |  |  |
| c(Water^#^) × c (Duration of exposure) | 0.057(0.063) |  |  |  |
| c(Water^#^) × c (Hukou status) | -0.083(0.059) |  |  |  |
| c(Water^#^) × c(Ethnicity) | 0.095(0.087) |  |  |  |
| c(Water^#^) × c (Household income) | 0.056(0.062) |  |  |  |
| c (Intersection density) × c(gender) |  | -0.016(0.041) |  |  |
| c (Intersection density) × c (Duration of exposure) |  | -0.161(0.049) ** |  |  |
| c (Intersection density) × c (Hukou status) |  | 0.067(0.049) |  |  |
| c (Intersection density) × c(Ethnicity) |  | 0.194(0.069) ** |  |  |
| c (Intersection density) × c (Household income) |  | -0.065(0.044) |  |  |
| c (Fast food restaurant density) × c (gender) |  |  | -0.002(0.004) |  |
| c (Fast food restaurant density) × c (Duration of exposure) |  |  | -0.005(0.004) |  |
| c (Fast food restaurant density) × c (Hukou status) |  |  | -0.001(0.004) |  |
| c (Fast food restaurant density) × c (Ethnicity) |  |  | -0.005(0.006) |  |
| c (Fast food restaurant density) × c (Household income) |  |  | -0.001(0.004) |  |
| c (Take-away sweets shops) × c (gender) |  |  |  | 0.006(0.006) |
| c (Take-away sweets shops) × c (Duration of exposure) |  |  |  | -0.005(0.007) |
| c (Take-away sweets shops) × c (Hukou status) |  |  |  | 0.008(0.007) |
| c (Take-away sweets shops) × c (Ethnicity) |  |  |  | -0.007(0.009) |
| c (Take-away sweets shops) × c (Household income) |  |  |  | -0.009(0.007) |
| **Goodness of model fitting:** |  |  |  |  |
| Sample size | 22,009 | 22,009 | 22,009 | 22,0009 |
| AIC score | 128,181.1 | 128164.6 | 128,128.7 | 128,181.3 |

NBEs: natural and built environments; AIC: AIC: Akaike Information Criterion

The variable started by “c” represented the variable were centered;

Data reported with coefficient (β) and standardized error (S.E.);

Effect estimates of NEs are reported per interquartile range increase; ** p<0.01; *p<0.05;

Models were adjusted by covariates including body mass index(kg/m^2^), tertiles of time spent weekly on physical activity for active travel, age and campus location.

Supplementary Table 5 Results of multivariate linear associations between undergraduates’ depression and exposure to NBEs within 2.5 km buffer zone with interaction effects of socioeconomics

| **Independent variables** | β1 (S.E) | β2 (S.E) | β3 (S.E) | β4 (S.E) |
| --- | --- | --- | --- | --- |
| **Natural environments** |  |  |  |  |
| NDVI^#^ | 0.073(0.039) | 0.051(0.039) | 0.064(0.039) | 0.065(0.039) |
| Dense trees^#^ | 0.055(0.041) | 0.067(0.041) | 0.061(0.041) | 0.053(0.041) |
| Scattered trees^#^ | 0.017(0.033) | 0.020(0.033) | 0.017(0.033) | 0.025(0.033) |
| Bush, scrub^#^ | 0.060(0.035) | 0.056(0.035) | 0.059(0.035) | 0.064(0.035) |
| Low plants^#^ | -0.040(0.038) | -0.038(0.038) | -0.044(0.038) | -0.044(0.038) |
| Water^#^ | -0.121(0.031) ** | -0.120(0.031) ** | -0.120(0.031) ** | -0.115(0.031) ** |
| **Built environments** |  |  |  |  |
| Population density (10000 population/km^2^) | 0.031(0.053) | 0.043(0.053) | 0.021(0.053) | 0.034(0.053) |
| Intersection density (10 intersections/km^2^) | -0.164(0.046) ** | -0.171(0.047) ** | -0.162(0.046) ** | -0.183(0.046) ** |
| Fast food restaurant density(restaurant/km^2^) | 0.009(0.004) * | 0.008(0.004) * | 0.010(0.004) ** | 0.009(0.004) * |
| Take-away sweets shops (shop/km^2^) | 0.014(0.004) ** | 0.014(0.004) ** | 0.015(0.004) ** | 0.014(0.004) ** |
| **Interaction effects with significant NBEs and individual attributes** |  |  |  |  |
| c (Water^#^) × c(gender) | -0.031(0.056) |  |  |  |
| c(Water^#^) × c (Duration of exposure) | -0.099(0.063) |  |  |  |
| c(Water^#^) × c (Hukou status) | -0.080(0.059) |  |  |  |
| c(Water^#^) × c(Ethnicity) | 0.128(0.083) |  |  |  |
| c(Water^#^) × c (Household income) | -0.069(0.063) |  |  |  |
| c (Intersection density) × c(gender) |  | -0.048(0.054) |  |  |
| c (Intersection density) × c (Duration of exposure) |  | -0.256(0.068) ** |  |  |
| c (Intersection density) × c (Hukou status) |  | 0.138(0.065) * |  |  |
| c (Intersection density) × c(Ethnicity) |  | 0.217(0.091) * |  |  |
| c (Intersection density) × c (Household income) |  | -0.045(0.059) |  |  |
| c (Fast food restaurant density) × c(gender) |  |  | 0.001(0.005) |  |
| c (Fast food restaurant density) × c (Duration of exposure) |  |  | -0.017(0.005) ** |  |
| c (Fast food restaurant density) × c (Hukou status) |  |  | -0.001(0.005) |  |
| c (Fast food restaurant density) × c (Ethnicity) |  |  | 0.0001(0.008) |  |
| c (Fast food restaurant density) × c (Household income) |  |  | -0.005(0.005) |  |
| c (Take-away sweets shops) × c (gender) |  |  |  | 0.006(0.008) |
| c (Take-away sweets shops) × c (Duration of exposure) |  |  |  | -0.016(0.009) |
| c (Take-away sweets shops) × c (Hukou status) |  |  |  | 0.018(0.009) * |
| c (Take-away sweets shops) × c (Ethnicity) |  |  |  | 0.002(0.013) |
| c (Take-away sweets shops) × c (Household income) |  |  |  | -0.008(0.009) |
| **Goodness of model fitting:** |  |  |  |  |
| Sample size | 22,009 | 22,009 | 22,009 | 22,009 |
| AIC score | 128,142.7 | 128,127.0 | 128,142.6 | 128,143.3 |

NBEs: natural and built environments;

The variable started by “c” represented the variable were centered;

Data reported with coefficient (β) and standardized error (S.E.);

Effect estimates of NEs are reported per interquartile range increase; ** p<0.01; *p<0.05;

Models were adjusted by covariates including body mass index(kg/m^2^), tertiles of time spent weekly on physical activity for active travel, age and campus location.

# Supplementary figures


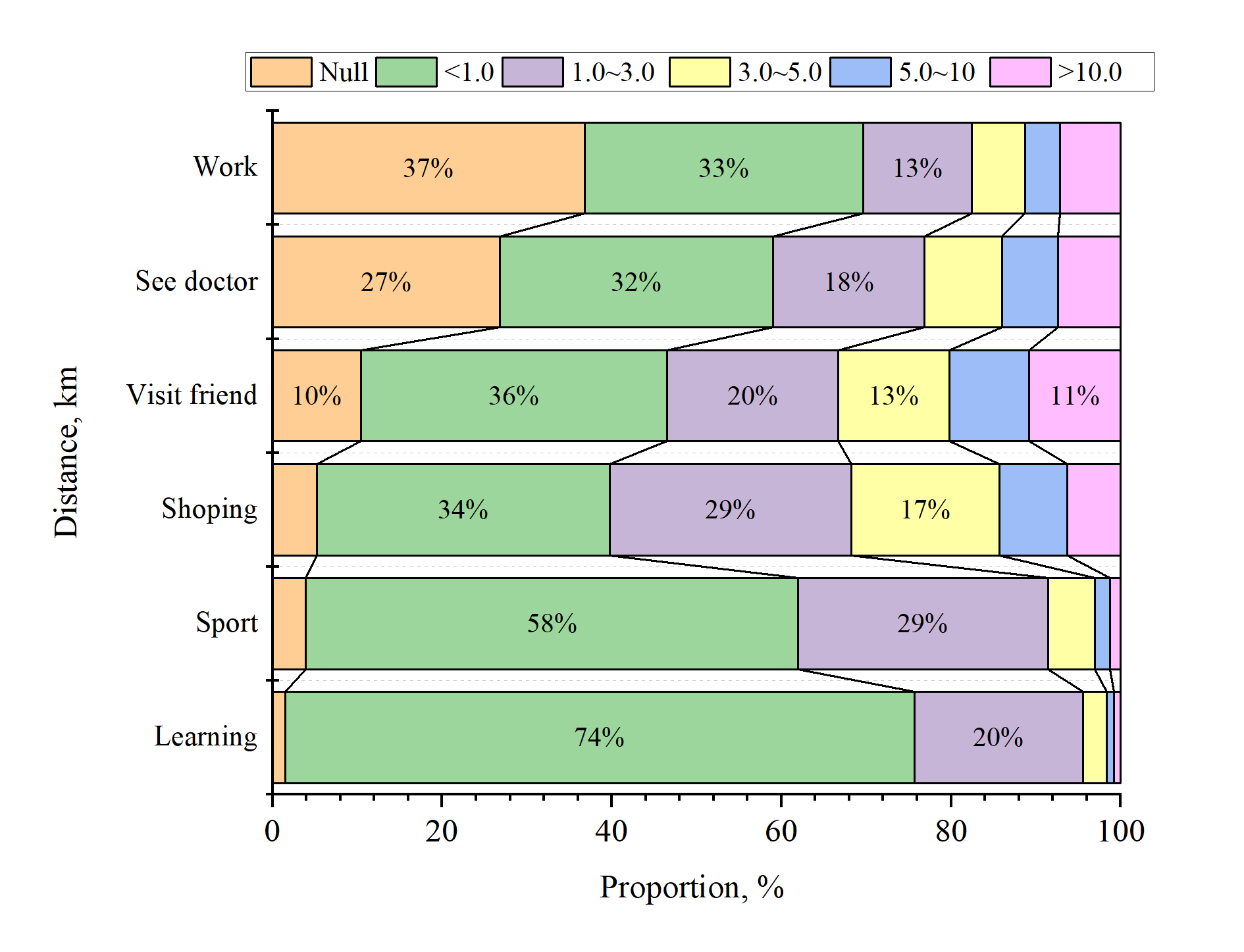


Supplementary Figure 1 Distribution of undergraduates’ transport distance from dormitory to their recreational and learning places. The labels were hide for the bars less than 10%.


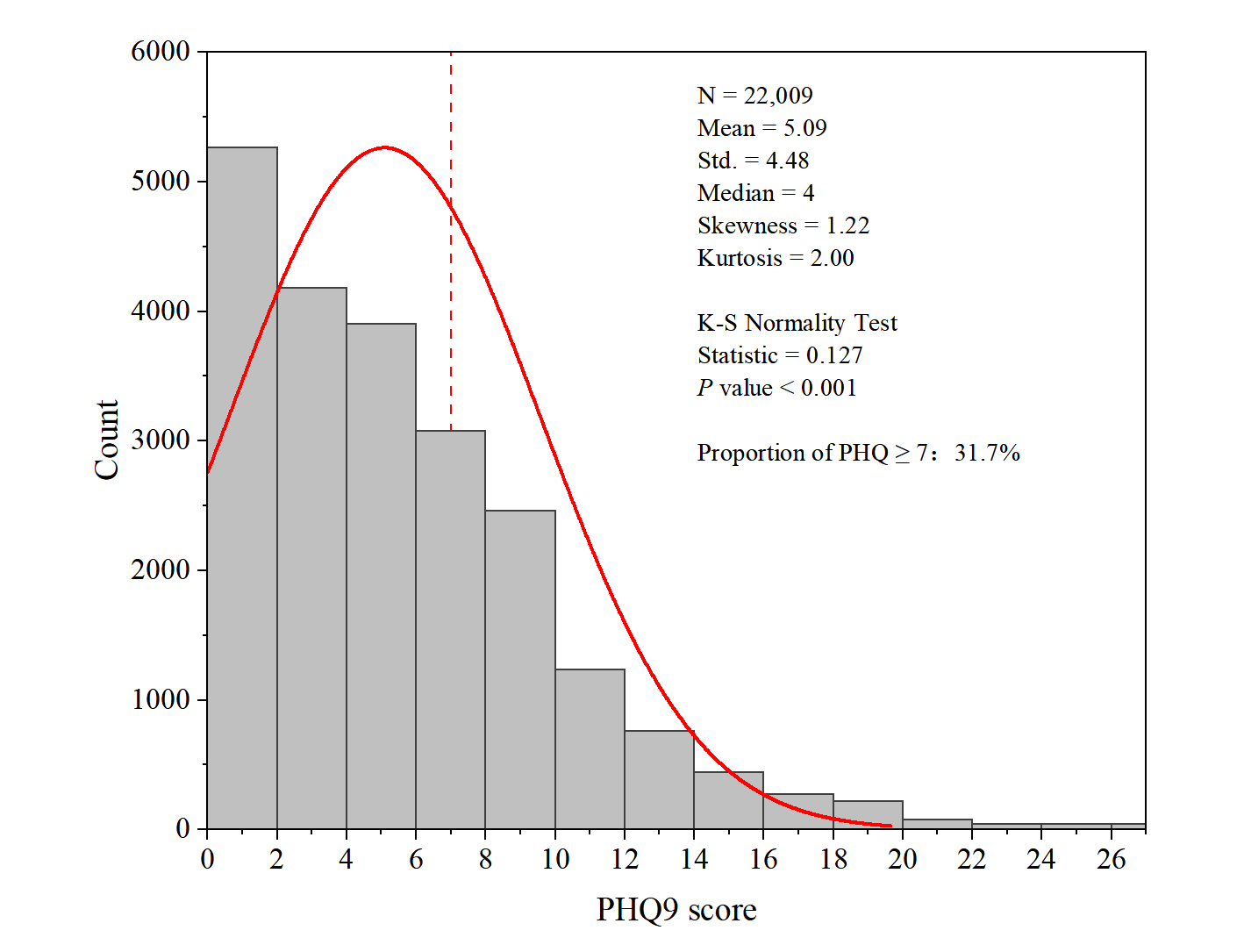


**Supplementary Figure 2 Distribution of PHQ9 score.**
